# Association between serum leptin concentrations and homeostasis model assessment-insulin resistance of 2.5 and higher in normal weight Japanese women

**DOI:** 10.1038/s41598-023-35490-0

**Published:** 2023-05-22

**Authors:** Satomi Minato-Inokawa, Yuuna Hayashida, Mari Honda, Ayaka Tsuboi-Kaji, Mika Takeuchi, Kaori Kitaoka, Miki Kurata, Bin Wu, Tsutomu Kazumi, Keisuke Fukuo

**Affiliations:** 1 Research Institute for Nutrition Sciences, Mukogawa Women’s University, 6-46, Ikebiraki-cho, Nishinomiya, Hyogo 663-8558 Japan; 2 Laboratory of Community Health and Nutrition, Department of Bioscience, Graduate School of Agriculture, Ehime University, Matsuyama, Ehime Japan; 3 Center for the Promotion of Interdisciplinary Education and Research, Kyoto University, Kyoto, Japan; 4 Department of Food Sciences and Nutrition, Mukogawa Women’s University, Nishinomiya, Hyogo Japan; 5 Open Research Center for Studying of Lifestyle-Related Diseases, Mukogawa Women’s University, Nishinomiya, Hyogo Japan; 6 Department of Health, Sports, and Nutrition, Faculty of Health and Welfare, Kobe Women’s University, Kobe, Hyogo Japan; 7 Department of Nutrition, Osaka City Juso Hospital, Osaka, Japan; 8 Department of Advanced Epidemiology, Noncommunicable Disease (NCD) Epidemiology Research Center, Shiga University of Medical Science, Otsu, Shiga Japan; 9 Department of Endocrinology, First Affiliated Hospital of Kunming Medical University, Kunming, Yunnan China; 10 Department of Medicine, Kohan Kakogawa Hospital, Kakogawa, Hyogo Japan

**Keywords:** Endocrinology, Medical research, Risk factors

## Abstract

Normal weight insulin resistant phenotype was characterized in 251 Japanese female university students using homeostasis model assessment-insulin resistance. Birth weight, body composition at age 20, cardiometabolic traits and dietary intake were compared cross-sectionally between insulin sensitive (< 1.6, n = 194) and insulin resistant (2.5 and higher, n = 16) women. BMI averaged < 21 kg/m^2^ and waist < 72 cm and did not differ between two groups. The percentage of macrosomia and serum absolute and fat-mass corrected leptin concentrations were higher in insulin resistant women although there was no difference in birth weight, fat mass index, trunk/leg fat ratio and serum adiponectin. In addition, resting pulse rate, serum concentrations of free fatty acids, triglycerides and remnant-like particle cholesterol were higher in insulin resistant women although HDL cholesterol and blood pressure did not differ. In multivariate logistic regression analyses, serum leptin (odds ratio:1.68, 95% confidential interval:1.08–2.63, *p* = 0.02) was associated with normal weight insulin resistance independently of macrosomia, free fatty acids, triglycerides, remnant-like particle cholesterol and resting pulse rate. In conclusion, normal weight IR phenotype may be associated with increased plasma leptin concentrations and leptin to fat mass ratio in young Japanese women, suggesting higher leptin production by body fat unit.

## Introduction

It is well-known that obesity and race/ethnicity are risk factors for type 2 diabetes^1^, characterized by β cell dysfunction and insulin resistance. It is also well-known that the relationship between body weight and diabetes risk may differ by race/ethnicity^2^. It is widely recognized that type 2 diabetes in East Asians is characterized primarily by β cell dysfunction, and less adiposity and hence insulin resistance compared to the disease in Caucasians^3^. For example, when homeostasis model assessment-insulin resistance (HOMA-IR)^4^, a most commonly used biomarker of insulin resistance, increases from normal glucose tolerance through impaired glucose tolerance to type 2 diabetes, fasting glucose concentrations increase while there was no change in BMI and fasting insulin not only in Japanese normal weight people but also in overweight people^5^. However, a recent study from Japan suggests that insulin resistance, in addition to β cell dysfunction, contributed strongly to the development of type 2 diabetes in a Japanese population^6^.

In Asian countries, type 2 diabetes is prevalent in people who are underweight or normal weight in addition to overweight people^7,8^. For example, we previously reported weight history since young adults in Japanese patients with type 2 diabetes^9^, in whom two-thirds of them were lean or normal weight at the time of the study with a mean BMI of 24.1 kg/m^2^). It is, therefore, important to characterize a normal weight but insulin resistant phenotype. We investigated this issue in young nondiabetic Japanese adults in the present study.

## Subjects and methods

We reanalyzed cross-sectionally 251 normal weight (BMI: 18.5–24.9 kg/m^2^) women, whose age averaged 20.6 years and BMI 20.6 kg/m^2^ among 307 young Japanese women whose details were reported previously^10^. They were students of Department of Food Sciences and Nutrition, Mukogawa Women's University, and were recruited as volunteers. Among 251 women, 166 and 181 women provided data on dietary intake and birthweight, respectively, the latter of which was obtained either through maternal health check notes or child health notebook records (issued by each municipal office). Women with clinically diagnosed acute or chronic inflammatory diseases, endocrine, cardiovascular, hepatic, renal diseases, hormonal contraception, unusual dietary habits were excluded from the study. This research followed the tenets of the Declaration of Helsinki. The study was approved by the Ethical Committees of Mukogawa Women’s’ University (No. 07–28, on 19/02/2008) and was in accordance with the Helsinki declaration. All participants gave written informed consent after the experimental procedure had been explained.

After a 12-h overnight fast, participants underwent blood sampling, measurements of anthropometric indices, blood pressure and body composition as previously described^10–13^. Blood pressure was measured using an automated sphygmomanometer (BP-203RV II, Colin, Tokyo, Japan) after participants were seated at least for 5 min. Plasma glucose, serum insulin, triglycerides, cholesterol, high-density lipoprotein (HDL) cholesterol, free fatty acid (FFA), HbA1c, leptin and adiponectin were quantified as previously reported^10–13^. Remnant-like particle (RLP) cholesterol was measured by the immunoseparation technique^14^ using a commercially available kit (Japan Immunoresearch Laboratories, Takasaki, Japan). Metabolic syndrome was defined according to the modified criteria of the National Cholesterol Education Program Adult Treatment Panel III guidelines^15^. The leptin to adiponectin ratio (LAR) was calculated as a marker of compromised adipose tissue function^16^.

HOMA-IR^4^ was calculated as a product of fasting insulin (μU/mL) and fasting glucose (mg/dL)/405. HOMA-IR < 1.6 was defined as normal and ≥ 2.5 as insulin resistant (IR)^17^. HOMA-IR between 1.6 and 2.4 was referred to as borderline state and HOMA-IR < 1.6 as insulin sensitive (IS) state instead of normal in the present study. Homeostasis model assessment-insulin secretion (HOMA-β) was calculated as well^4^.

A standard 75 g oral glucose tolerance test (OGTT) was done in 99 women. The blood was withdrawn at 0 (fasting), 30 min, 1 h, and 2 h for glucose and insulin measurements. The area under the response curve of glucose (AUCg) and serum insulin (AUCi) was calculated by the trapezoidal method.

Whole-body dual-energy X-ray absorptiometry (DXA) (Hologic QDR-2000 software version 7.20D, Waltham, MA) was used to measure lean tissue mass, fat mass, and bone mineral mass for arms, legs (lower-body), trunk and the total body^12^. General adiposity was assessed using BMI and body fat mass index (FMI), the latter was calculated as body fat mass in kg divided by height in meter squared. Trunk and leg FMI were calculated as well. Waist circumference and the ratio of trunk to leg fat^18^ were considered as markers of abdominal fat accumulation. Muscle characteristics were evaluated by relative appendicular skeletal muscle mass (ASM) as percentage of body mass (%ASM) and absolute ASM index (ASM/height^2^ in kg/m^2^). %ASM is suggested to be a better predictor of insulin resistance and diabetes risk than ASM or ASM index^19^.

Dietary intake of the previous month was assessed using the self-administered diet history questionnaire^20^. This has been widely used throughout Japan and its validity with respect to commonly studied nutrition factors has been confirmed.

Data were presented as mean ± SD unless otherwise stated. The leptin to fat mass ratio was computed as previously described^21^. Differences among three groups were analyzed by analysis of variance and then Bonferroni's multiple comparison procedure. Stepwise multivariate logistic regression analyses were used to identify most important determinants of IR state. Independent variables included were variables which showed significant difference among three groups. Correlations between anthropometric and metabolic variables were investigated by Pearson’s analysis. A two-tailed *p* < 0.05 was considered statistically significant. All calculations were performed with SPSS system 23.0 (SPSS Inc, Chicago, IL).

### Ethical approval

The study was approved by the Ethics Committees of the Mukogawa Women’s University (No. 07–28 on 19/02/2008).

## Results

BMI averaged 20.6 ± 1.4 kg/m^2^ and HOMA-IR 1.22 ± 0.76 in a total population. IR was present in 16 women (6.4%) while 194 women (77.3%) were insulin sensitive (Table 1). However, BMI averaged 20.6 kg/m^2^, waist 71.0 cm and ALT 11 U/L in IR women. Of 251 normal weight Japanese women, 239 (95.2%) had none of metabolic syndrome components, 11 (4.4%) had a single component and nobody had metabolic syndrome. Only one normal weight woman had 2 components.

**Table 1:** Birthweight, current body composition, serum liver enzymes and adipocytokines in young normal weight Japanese women according to homeostasis model assessment-insulin resistance values: insulin sensitive (IS), borderline and insulin resistant (IR) groups.

HOMA-IR: range	IS	Borderline	IR	*
	n = 194	n = 41	n = 16	
	< 1.6	1.6–2.4	≥ 2.5	
Birthweight (g)	3168 ± 374	3285 ± 356	3366 ± 631	
Macrosomia (n, %)	4, 2.8	2, 7.1	2, 22.2	0.016
BMI (kg/m^2^)	20.5 ± 1.5	20.6 ± 1.3	20.6 ± 1.5	
Waist (cm)	71.5 ± 5.1	71.3 ± 4.6	71.0 ± 5.1	
Trunk/leg fat ratio	1.26 ± 0.23	1.25 ± 0.27	1.32 ± 0.28	
% Body fat (%)	27.9 ± 4.6	29.6 ± 4.5	29.6 ± 4.7	
%Trunk fat (%)	28.9 ± 5.4	30.3 ± 5.5	30.8 ± 6.1	
%ASM (%)	28.9 ± 2.0	28.1 ± 2.1	28.5 ± 2.5	
ASMI (kg/m^2^)	5.9 ± 0.5	5.8 ± 0.4	5.9 ± 0.5	
Body FMI (kg/m^2^)	5.7 ± 1.3	6.0 ± 1.2	6.1 ± 1.3	
Trunk FMI (kg/m^2^)	2.76 ± 0.70	2.91 ± 0.71	3.00 ± 0.86	
Leg FMI (kg/m^2^)	2.21 ± 0.47	2.35 ± 0.45	2.27 ± 0.35	
Leptin (ng/mL)	8.1 ± 3.2	10.1 ± 4.3	11.4 ± 5.0	a,b
Fat mass-adjusted leptin (ng/mL/kg)	0.56 ± 0.17	0.65 ± 0.18	0.73 ± 0.24	a,b
Adiponectin (µg/mL)	11.4 ± 4.0	11.4 ± 5.0	11.2 ± 3.6	
LAR	0.82 ± 0.53	1.12 ± 0.89	1.19 ± 0.79	a
Systolic BP (mmHg)	106 ± 9	106 ± 13	107 ± 7	
Diastolic BP (mmHg)	61 ± 7	61 ± 8	61 ± 7	
Resting pulse (bpm)	63 ± 8	71 ± 11	70 ± 11	a,b

Mean ± SD or n, %. HOMA-IR: homeostasis model assessment-insulin resistance, BMI: body mass index, ASM: appendicular skeletal muscle mass, ASMI: ASM index, %ASM: percentage ASM, FMI: fat mass index, BP: blood pressure. bpm: beats per minute. LAR: leptin/adiponectin ratio. *: *p* < 0.05 or less by Bonferroni's multiple comparison procedure. a: IS versus borderline, b: IS versus IR, c: borderline versus IR.

**Figure 1:**
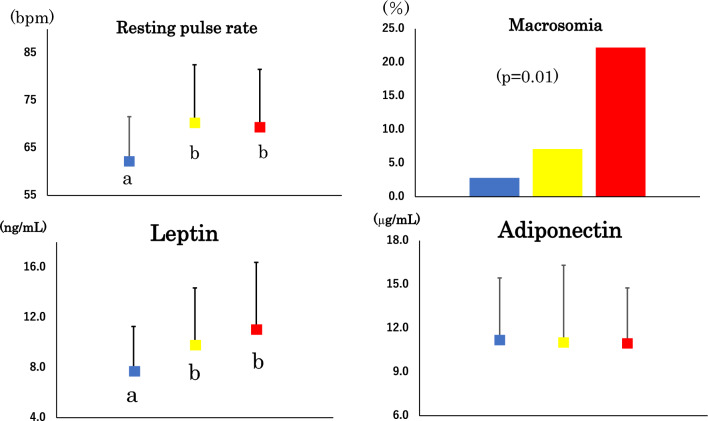
Percentage of macrosomia, resting pulse rate, serum leptin and adiponectin in young normal-weight Japanese women who were insulin sensitive (n = 194), borderline (n = 41) and insulin resistant (n = 16) (blue, yellow and red bar or squares, respectively). Mean ± SD. Means not sharing common alphabetical letter are significantly different with each other at *p* < 0.05 or less by Bonferroni’s multiple comparison procedure.

The prevalence of macrosomia was higher in IR women whereas there was no difference in birthweight (Table 1 and Fig. 1). Resting pulse rate was higher in borderline and IR compared to IS but blood pressure did not differ. Although there was no statistical difference in BMI, waist, %BF, trunk-to-leg fat ratio, body, trunk and leg FMI, ASMI and %ASM, serum leptin and fat-mass adjusted leptin were higher in borderline and IR than in IS. Although serum adiponectin did not differ LAR was higher in borderline women and tended to be higher in IR women (*p* = 0.07) than in IS women. When compared between women with HOMA-IR < 1.6 and those with ≥ 1.6 (Supplementary  Table 1), birthweight, prevalence of macrosomia, and FMI were tended to be higher and ASMI was tended to be lower in women with HOMA-IR ≥ 1.6.

Body FMI showed positive associations with fasting insulin (r = 0.15, *p* = 0.02), HOMA-IR (r = 0.13, *p* = 0.04), leptin (r = 0.65, *p* < 0.001), fat-mass adjusted leptin (r = 0.19, *p* = 0.003) and LAR (r = 0.47, *p* < 0.001). In contrast, ASMI showed no association with these variables.

**Figure 2:**
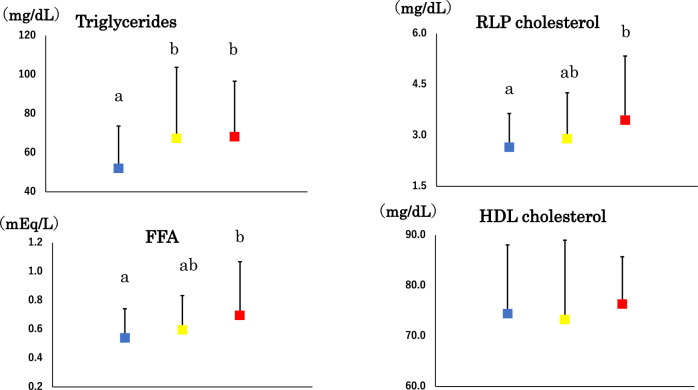
Serum triglycerides, free fatty acid (FFA), high-density lipoprotein (HDL) and remnant-like particle (RLP) cholesterol in young normal-weight Japanese women who were insulin sensitive (blue squares), borderline (yellow squares) and insulin resistant (red squares). Mean ± SD. Means not sharing common alphabetical letter are significantly different with each other at *p* < 0.05 or less by Bonferroni’s multiple comparison procedure.

As HOMA-IR increased, so did fasting glucose, insulin, FFA and HOMA-β in a stepwise fashion (Table 2). However, FFA increased by 38% whereas fasting glucose increased by 10%. Despite a small number of OGTT in IR (n = 3), 2-h insulin and AUCi were significantly higher in IR than in IS while differences in 2-h glucose and AUCg were not significant. Serum triglycerides, RLP cholesterol and FFA were higher in IR than in IS (Table 2 and Fig. 2). There was no difference in HbA1c and HDL cholesterol.

**Table 2:** Glucose and lipid metabolism in young normal weight Japanese women according to homeostasis model assessment-insulin resistance values: insulin sensitive (IS), borderline and insulin resistant (IR) groups.

	IS	Borderline	IR	*
Fasting glucose (mg/dL)	81 ± 6	86 ± 6	89 ± 9	a,b
2-h glucose (mg/dL)^#^	92 ± 24	92 ± 14	117 ± 20	
Fasting insulin (μU/mL)	5 ± 2	9 ± 2	14 ± 4	a,b,c
2-h insulin (μU/mL)^#^	37 ± 24	49 ± 11	98 ± 25	b,c
HbA1c (%)	5.2 ± 0.2	5.2 ± 0.3	5.2 ± 0.3	
HOMA-β	103 ± 67	162 ± 65	257 ± 248	a,b,c
HOMA-IR	0.9 ± 0.4	2.0 ± 0.3	3.1 ± 0.9	a,b,c
AUCg (mg/dL/2 h)^#^	204 ± 43	199 ± 37	251 ± 56	
AUCi (μU/mL/2 h)^#^	73 ± 32	130 ± 54	146 ± 22	a,b
Triglycerides (mg/dL)	53 ± 21	67 ± 37	68 ± 29	a,b
Cholesterol (mg/dL)	182 ± 26	184 ± 29	188 ± 25	
HDL-C (mg/dL)	75 ± 13	73 ± 16	77 ± 9	
RLP-C (mg/dL)	2.7 ± 0.9	3.0 ± 1.2	3.6 ± 1.7	b
FFA (mEq/L)	0.53 ± 0.21	0.61 ± 0.23	0.73 ± 0.34	b

Mean ± SD, ^#^: n = 89, 7 and 3 in IS, borderline and IR, respectively, HOMA-IR and HOMA-β: homeostasis model assessment-insulin resistance and secretion, respectively, AUCg and AUCi: area under the response curve of glucose and insulin, respectively, HDL-C: high-density lipoprotein cholesterol, RLP-C: remnant-like particle cholesterol, FFA: free fatty acid. *: the same as in Table 1.

There was no difference in daily energy intake and macronutrients among three groups of normal weight women (data not shown). There was no difference in daily intake of saturated fat, monounsaturated fat and polyunsaturated fat.

In multivariate logistic regression analyses, which included triglycerides, RLP cholesterol, FFA, leptin and pulse rate as independent variables, serum leptin emerged as a single determinant of normal weight IR (odds ratio:1.68, 95% confidential interval:1.08–2.63, *p* = 0.02).

## Discussion

The current study demonstrated that HOMA-IR ≥ 2.5 was associated with serum leptin and fat-mass adjusted leptin concentrations in young normal weight Japanese women. It was also associated with FFA, triglycerides and RLP cholesterol, the last of which is an independent predictor of cardiovascular disease in women^22^. Further, HOMA-IR ≥ 2.5 was associated with higher resting pulse rate. These associations were independent of FMI and trunk/leg fat ratio, sophisticated measures of body fat mass and abdominal fat accumulation, respectively, despite remarkable differences in HOMA-IR. Among these variables, serum leptin or fat-mass adjusted leptin concentrations emerged as an independent determinant of normal weight IR. It is noteworthy that waist and ALT averaged 71.0 cm and 11.3 U/L, respectively, in women with normal weight IR, suggesting a minimum abdominal and hepatic fat accumulation, respectively.

Studies on association between HOMA-IR and leptin in normal weight individuals were limited although significant association was reported in multiple studies in the general population and in diabetic patients^23–27^. We found two studies: one in European children^28^ and the other in a general population in China^29^. Both studies found a significant and independent association between HOMA-IR and leptin in normal weight people. In the present study, normal weight IR women had higher leptin levels, leptin to fat mass ratio and LAR, a marker of adipose tissue dysfunction^16^. As adipocyte hypertrophy, another marker of adipose tissue dysfunction, has been shown to be associated with increased plasma leptin concentrations^30^, elevated leptin concentrations in IR women might be related to higher leptin production by body fat unit.

FMI showed positive associations with fasting insulin and leptin in the present study. We^31^ previously reported in 481 college female students, in whom 251 normal weight women in the present study were included that after mutual adjustment both trunk and leg fat mass showed positive associations with leptin whereas trunk fat mass was negatively, and leg fat mass was positively associated with adiponectin. Trunk fat showed positive associations with fasting and 2 h-insulin whereas leg fat showed negative associations with 2 h-insulin but no association with fasting insulin^31^.

As HOMA-IR increased, so did HOMA-β in a stepwise fashion. Therefore, 2-h glucose, AUCg and HbA1c did not differ significantly among three groups. Although 2-h glucose and AUCg were somewhat higher in IR women, there was no difference between women with HOMA-IR < 1.6 and ≥ 1.6 (Supplementary   Table 1). Normal weight IR women had higher 2-h insulin than the other two groups of women in the present study. This may be in line with a study in normal-weight, Hispanic women which suggested that 2-h insulin in addition to HOMA-IR and fasting insulin may be an important clinical marker for insulin resistance^32^.

Hypertriglyceridemia is a common lipid abnormality in people with visceral obesity, metabolic syndrome and type 2 diabetes^33^. Insulin resistance is often an underlying feature and results in increased FFA delivery to the liver due to increased peripheral lipolysis. Increased FFA flux to the liver results in hypertriglyceridemia, which may be associated with higher RLP cholesterol^34^. These findings may be in line with our observation that women with HOMA-IR ≥ 2.5 had increased FFA, triglycerides and RLP cholesterol.

Increased FFA in IR women may be associated with adipose tissue insulin resistance, which can be evaluated by adipose tissue insulin resistance (AT-IR) index^35^, which was calculated as a product of fasting insulin (μU/mL) and FFA (mEq/L). AT-IR increased in a stepwise fashion from IS through borderline to IR women in the present study (2.5 ± 1.4, 6.3 ± 2.7 and 10.1 ± 5.1, respectively, all *p* < 0.001). Elevated FFA in IR women also might be related to adipocyte hypertrophy as fat cell size was reported to be correlated positively with lipolysis and HOMA-IR in non-obese individuals with type 2 diabetes^36^.

The percentage of IR (HOMA ≧ 2.5) was similar in normal weight middle-aged Japanese people^37^ and young Japanese women in the present study (6.2 vs. 6.4%) although the percentage of IS was somewhat smaller in middle-aged adults (67.5 vs. 77.3%).

The accurate and reliable measures of general and central fat accumulation by DXA are the strength of the present study. There are several limitations of this study including the cross-sectional design, relatively small sample size, and a single measurement of biochemical variables. The study population included only female university students. However, this also might be considered as a strength since it eliminates potential confounding factors^12^. For example, more than 90% of grade 1 students are 18 years old. This may decrease the interference of age and environmental factors, including smoking, alcohol, educational, and socioeconomic status. Further, in almost all students, almost all school expenses were covered by parents, suggesting that socioeconomic status appears to be less heterogeneous among parents who fed participants of the present study. We did not assess family history of obesity and current exercise habits. However, although they were not engaged in any regular sport activity participants had 9367 ± 1971 steps/day (mean ± SD of 77 participants). Statistical power and sample size were not calculated. As participants were young Japanese women, the results may not be generalized to other gender, age populations, races or ethnicities.

In conclusion, normal weight IR phenotype may be associated with increased plasma leptin concentrations and leptin to fat mass ratio in young Japanese women, suggesting higher leptin production by body fat unit. Prospective follow-up studies are needed to see if these phenotypes predict clinical outcomes (i.e., development of diabetes or cardiovascular disease).

## Supplementary Information

Supplementary Table 1.

## Data Availability

The datasets used and/or analyzed during the current study available from the corresponding author on reasonable.
