# Supplementary material for: Association between serum leptin concentrations and homeostasis model assessment-insulin resistance of 2.5 and higher in normal weight Japanese women
Source: Sci Rep. 2023 May 22;13:8217. doi: 10.1038/s41598-023-35490-0 (PMC10203141; doi:10.1038/s41598-023-35490-0)
Supplement: Supplementary file 1 — Supplementary Table 1. [file 41598_2023_35490_MOESM1_ESM.docx]

| Supplementary table 1. Comparison between women with homeostasis model assessment-insulin resistance<1.6 and ≥1.6 | | | | | | | |
| --- | --- | --- | --- | --- | --- | --- | --- |
| HOMA-IR | <1.6 | | | ≥1.6 | | |  |
|  | n=194 | | | n=57 | | | p values |
| Birthweight (g) | 3168 | ± | 374 | 3305 | ± | 430 | 0.056 |
| Macrosomia (n, %) | 4 | , | 2.8 | 4 | , | 10.8 | 0.055 |
| BMI (kg/m2) | 20.5 | ± | 1.5 | 20.6 | ± | 1.3 | 0.801 |
| Waist (cm) | 71.5 | ± | 5.1 | 71.2 | ± | 4.6 | 0.742 |
| Trunk/leg fat ratio | 1.26 | ± | 0.23 | 1.27 | ± | 0.27 | 0.826 |
| % Body fat (%) | 27.9 | ± | 4.6 | 29.6 | ± | 4.5 | 0.016 |
| %Trunk fat (%) | 28.9 | ± | 5.4 | 30.4 | ± | 5.6 | 0.059 |
| %ASM (%) | 28.9 | ± | 2.0 | 28.2 | ± | 2.2 | 0.032 |
| ASMI (kg/m2) | 5.92 | ± | 0.46 | 5.79 | ± | 0.45 | 0.070 |
| Body FMI (kg/m2) | 5.69 | ± | 1.25 | 6.06 | ± | 1.21 | 0.051 |
| Trunk FMI (kg/m^2^) | 7.00 | ± | 1.81 | 7.39 | ± | 1.94 | 0.161 |
| Leg FMI (kg/m^2^) | 2.21 | ± | 0.47 | 2.33 | ± | 0.42 | 0.091 |
| 2-h glucose (mg/dL) | 92 | ± | 24 | 99 | ± | 19 | 0.330 |
| AUCg (mg/dL/2h) | 204 | ± | 43 | 214 | ± | 47 | 0.471 |
| Leptin (ng/mL) | 8.1 | ± | 3.2 | 10.5 | ± | 4.5 | <0.001 |
| Fat mass-adjusted leptin (ng/mL/kg) | 0.56 | ± | 0.17 | 0.67 | ± | 0.20 | <0.001 |
| Adiponectin (µg/mL) | 11.4 | ± | 4.0 | 11.3 | ± | 4.6 | 0.868 |
| Leptin/adiponectin ratio | 0.82 | ± | 0.53 | 1.14 | ± | 0.86 | 0.010 |
| Systolic BP (mmHg) | 106 | ± | 9 | 106 | ± | 11 | 0.699 |
| Diastolic BP (mmHg) | 61 | ± | 7 | 61 | ± | 8 | 0.688 |
| Resting pulse (bpm) | 63 | ± | 8 | 71 | ± | 11 | <0.001 |
| Mean ± SD or n, %. Abbreviations are the same as in Tables 1 and 2. | | | | | | | |
